# Highly Sustainable and Completely Amorphous Hierarchical Ceramide Microcapsules for Potential Epidermal Barrier

**DOI:** 10.3390/polym12092166

**Published:** 2020-09-22

**Authors:** Joonsik Yoon, Minjoo Noh, Jun Bae Lee, Jun Hyup Lee

**Affiliations:** 1 Department of Chemical Engineering, Myongji University, Yongin 17058, Korea; sky4454278@naver.com; 2 Innovation Lab, Cosmax R&I Center, Cosmax, Seongnam 13486, Korea; mj1010@cosmax.com (M.N.); jblee@cosmax.com (J.B.L.); 3 Department of Chemical Engineering, Soongsil University, Seoul 06978, Korea

**Keywords:** amorphous structure, ceramide, critical packing parameter, epidermal barrier, polymer microcapsule

## Abstract

As a main component of the stratum corneum, ceramides can construct protective lamellae to provide an epidermal barrier against dehydration or external microorganisms. However, as ceramide molecules can easily form the isolated crystalline phase through self-assembly due to the amphipathic nature of bioactive lipids, the effective incorporation of ceramides into liquid media is the remaining issue for controlled release. Here, we report an unprecedented effective strategy to fabricate a completely amorphous and highly sustainable hierarchical ceramide polymer microcapsule for promising epidermal barrier by using the interpenetrating and cooperative self-construction of conical amphiphiles with a different critical packing parameter. The self-constructed amorphous architecture of ceramides in polymer microcapsule is achieved by the facile doping of conical amphiphiles and subsequent in situ polymerization of shell polymer in the core-shell geometry. It is experimentally revealed that an irregular cooperative packing structure formed by adaptive hydrophobic–hydrophilic interactions of cylindrical ceramides and conical amphiphiles in the confined microcapsule geometry enables a completely amorphous morphology of ceramides to be realized during the spontaneous encapsulation process. Furthermore, this elegant approach affords a highly dispersible and uniform hierarchical amorphous ceramide microcapsule with a greatly enhanced long-term stability compared to conventional crystalline ceramides.

## 1. Introduction

Human and animal skin plays an important role in the prevention of dehydration and temperature control, as well as in protection against physical, chemical, and biological attacks. Most of these functions are fulfilled by the skin lipids of stratum corneum, located in the outermost layers of the epidermis [1,2,3]. Among the components of skin lipids, the highest content of ceramides comprises more than 40% of the skin lipids produced in the body. Skin lipid mixtures have amphipathic properties due to their molecular structures, and thus these amphiphilic lipid molecules can form a dense brick-like structure, which is beneficial for maintaining moisture in the body and serving as an external barrier through intermolecular interactions [4,5]. This robust lamellar architecture acts as a barrier to the skin, protecting it from the environment, and preventing dehydration [6]. Ceramide molecules, which occupy a high proportion of the stratum corneum skin barrier, significantly decrease in amount maintained and production in the human body as aging progresses, leading to weakening of the skin barrier functions [7,8]. This weakening lowers the dehydration prevention ability of the stratum corneum, inhibits surface protection from external stimuli, and causes various skin diseases, skin wrinkles, cracking, and the like. For these reasons, ceramides have been considered as an important component of new pharmaceuticals and cosmetics in recent years. In fact, moisturizer with ceramides, when applied to the skin, have been shown to have excellent moisturizing, anti-aging, and soothing effects [5].

The importance of ceramides is well known, but their stable and uniform incorporation into the existing medical or cosmetic liquid media remains difficult. Although the amphipathic properties of ceramides as bioactive lipids play a key role in forming an epidermal barrier with a highly robust lamellar structure, recrystallization or significant agglomeration of ceramides frequently occur in liquid media due to the formation of the isolated crystalline phase through self-assembly. As a consequence, the poor solubility and dispersibility in liquid media make it difficult to introduce the efficacy of ceramides for potential epidermal barrier [9,10].

Microcapsules, which are microsized molecular carriers for wrapping functional materials in the core region, are widely applied in the food, pharmaceutical, printing, and cosmetic industries [11,12,13,14,15]. A wide variety of polymeric materials involving water-soluble gelatin, casein protein, and synthetic vinyl polymers is employed as encapsulation shells for microcapsules [11,12,13]. These polymeric microcapsules perform essential functions for the controlled release of core materials or localized selective reactions inside the spherical microreactors [16]. Among the various capsule shell materials, poly(methyl methacrylate) (PMMA) is considered suitable for medical and cosmetic applications because of its high chemical safety, biocompatibility, non-toxicity, and suitable mechanical properties [17,18]. The main purpose of PMMA microcapsules is to stably protect the inner core materials from the outside environment and to control the physical properties and release of the inner material [16]. Even though this microencapsulation approach could serve as a solution to suppress the severe agglomeration of core materials in the liquid media, thus far, the encapsulation of ceramides using polymeric microcapsules has not yet been reported in the literature.

**Figure 1:**
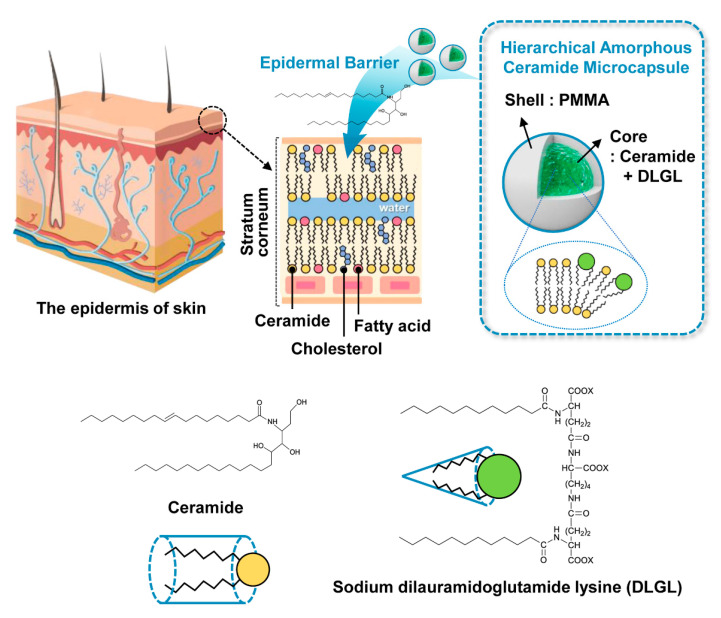
Schematic illustration of the fabrication of hierarchical amorphous ceramide microcapsules for potential epidermal barrier.

Herein, we present an unprecedented yet simple, effective, scalable strategy for the fabrication of completely amorphous and highly sustainable hierarchical ceramide microcapsules for potential epidermal barrier based on the interpenetrating and cooperative self-construction of conical amphiphiles with a different critical packing parameter in the confined geometry of polymeric microcapsule (Figure 1). The inherent concept which can construct the self-assembled hyperstructure of amphiphilic molecules depending on the molecular geometry is applied to the fabrication of the self-constructed amorphous architecture of ceramides in polymer microcapsule [19]. Notably, novel hierarchical amorphous ceramide microcapsule containing the interpenetrated cylindrical ceramide and conical sodium dilauramidoglutamide lysine (DLGL) as core materials could be facilely prepared in a large quantity using simple doping of DLGL amphiphile and subsequent in situ polymerization of PMMA shell in the core-shell geometry. The adaptive hydrophobic–hydrophilic interactions of cylindrical ceramides and conical DLGL amphiphiles enable an irregular cooperative packing structure inside the hierarchical PMMA microcapsule, thereby providing a completely amorphous morphology of ceramides during the spontaneous encapsulation process. In addition, excellent long-term durability of amorphous ceramide microcapsules with high dispersibility in the liquid media could be achieved compared to that of common crystalline ceramides.

## 2. Materials and Methods

### 2.1. Materials

Methyl methacrylate (MMA), sodium nitrite, and poly(vinyl alcohol) (PVA, *M*_n_ = 124,000–186,000) were purchased from Sigma Aldrich (Seoul, South Korea). The MMA monomer was purified to remove the polymerization inhibitors. Ethylene glycol dimethacrylate (EGDMA) was purchased from Acros (Pittsburgh, PA, USA). 2,2-Azobisisobutyronitrile (AIBN) was purchased from Junsei Chemicals (Tokyo, Japan) and used after purification. Methylene chloride (MC) and benzene used as dispersion solvents were purchased from Daejung Chemicals & Metals (Siheung, South Korea). Ceramide (Y-30) and sodium dilauramidoglutamide lysine (DLGL, Pellicer L-30) were obtained from Cosmax (Seongnam, Korea).

### 2.2. Synthesis of Hierarchical Amorphous Ceramide Microcapsule

First, 22.5 mL of a 1.5 wt% PVA solution was prepared in deionized (DI) water. PVA acts as a dispersion stabilizer to maintain spherical particles when synthesizing amorphous ceramide microcapsules via in situ suspension polymerization. 50 mg of ceramide and 50 mg of DLGL were dissolved in 2.5 mL of MC and 2.5 mL of benzene, respectively, and then the combined MC and benzene solution were stirred at 1000 rpm and 35 °C for 30 min. Then, 1.4 g of MMA, 0.002 g of EGDMA, and 0.02 g of AIBN were added to the combined solution, followed by stirring at 1000 rpm and 35 °C for 30 min. Next, this mixture was added to the PVA aqueous solution and stirred at 1000 rpm and 25 °C for 5 min. After stirring, 0.0025 g of sodium nitrite was added, and subsequently, the solution was polymerized in a two-necked flask equipped with a reflux condenser and nitrogen injector. Polymerization was carried out by stirring at 200 rpm and 80 °C for 9 h under a nitrogen atmosphere. Finally, the solution was purified with DI water and ethanol using a centrifuge at 10,000 rpm for 30 min at room temperature. The final product was dried in a vacuum oven at room temperature for 24 h. To investigate the effect of DLGL amphiphile on the self-constructed hyperstructure of ceramides in the core of polymer microcapsule, PMMA microcapsule containing only ceramide molecules was prepared according to the above synthetic procedure.

### 2.3. Characterization

The structure and size of hierarchical ceramide microcapsules were analyzed by Fourier-transform infrared (FT-IR, model: Jasco, FT-IR 460 Plus, Easton, PA, USA) spectrometer equipped with an attenuated total reflection (ATR) accessory, field emission scanning electron microscope (FE-SEM, model: Hitachi, SU-70, Tokyo, Japan), and energy dispersive X-ray spectroscopy (EDS, model: Horiba, Energy X-MaxN, Tokyo, Japan). The dispersion properties and morphology of the ceramide microcapsules were examined using an optical microscope (OM, model: Olympus, BX-51, Tokyo, Japan) and polarized optical microscope (POM, model: Olympus, BX-51, Tokyo, Japan). The morphological structure and crystallinity of the fabricated microcapsules were investigated by X-ray diffraction (XRD, model: Malvern Panalytical, AERIES, Malvern, United Kingdom) and differential scanning calorimetry (DSC, model: TA Instruments, DSC Q-20, New Castle, DE, USA). The DSC thermograms were obtained at a heating rate of 10 °C min^−1^ under nitrogen atmosphere. Thermogravimetric analysis (TGA, model: TA Instruments, SDT Q-600, New Castle, DE, USA) was performed from 30 to 700 °C under nitrogen flow.

## 3. Results and Discussion

### 3.1. Structural Characterization of Hierarchical Amorphous Ceramide Microcapsule

**Figure 2:**
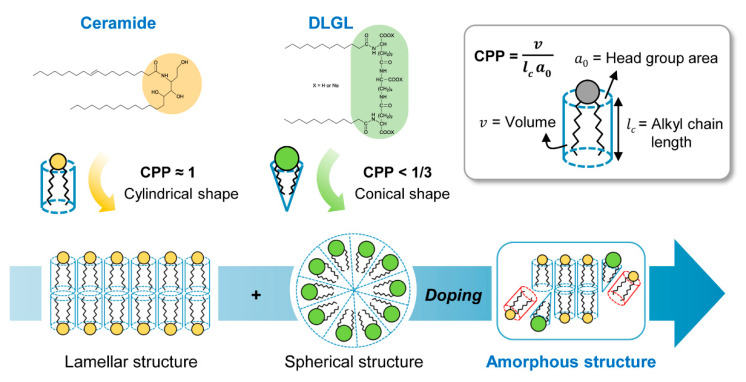
Schematic diagram of the self-constructed amorphous phase with an irregular cooperative packing structure.

The self-organized structure of amphiphilic molecules is mainly governed by critical packing parameter (CPP) derived from the head group area (*a*_o_) at the hydrophobic–hydrophilic interface, the length (*l*_c_) of the hydrophobic alkyl chain, and the volume (*v*) of molecule (Figure 2) [19]. The molecular structure of ceramide has been found to be cylindrical (CPP ≈ 1) through CPP calculations, which contributes to the formation of a highly crystalline lamellar structure [20,21,22,23,24]. A large number of cylindrical ceramide amphiphiles associate parallel to each other in the liquid medium through hydrophobic–hydrophilic interactions, resulting in the formation of substantial bilayer architecture. To break up the regular lamellar structure of ceramide molecules inducing the severe agglomeration or recrystallization in the medium, sodium dilauramidoglutamide lysine, which has a similar molecular structure to that of ceramide but a conical shape with a different critical packing parameter (CPP < 1/3), is employed as an amphiphilic dopant [25]. It is expected that conical DLGL amphiphiles can be interpenetrated into a cluster of cylindrical ceramide molecules through adaptive hydrophobic–hydrophilic interactions, leading to the formation of the self-constructed amorphous phase with an irregular cooperative packing structure in the confined microcapsule geometry [26]. In addition, the encapsulated inner mixture of ceramide and DLGL molecules can improve the penetration flux of ceramide for the epidermal barrier due to the chemical enhancer characteristic of biodegradable DLGL amphiphile [27].

**Figure 3:**
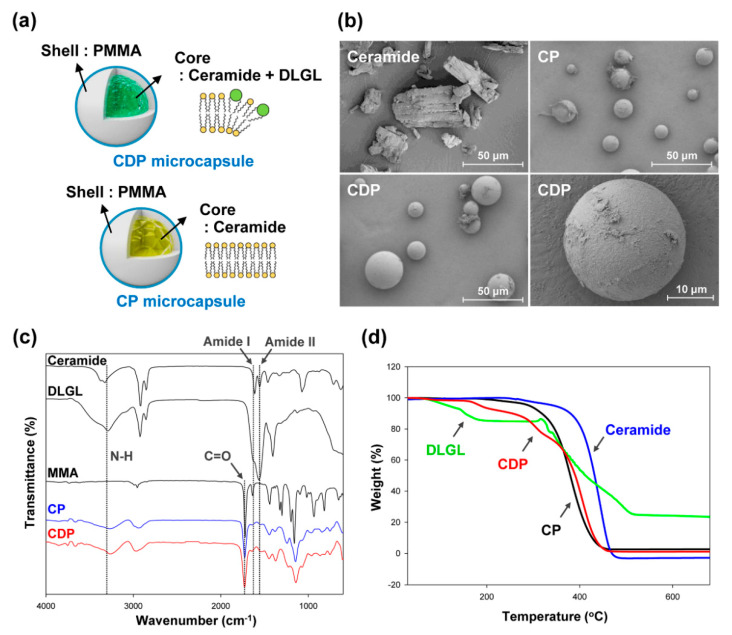
(**a**) Structures of CP and CDP microcapsules. (**b**) FE-SEM images of pure ceramide, CP microcapsule, and CDP microcapsule. (**c**) FT-IR spectra of pristine ceramide, pure DLGL, MMA monomer, CP microcapsule, and CDP microcapsule. (**d**) TGA thermograms of pristine ceramide, pure DLGL, CP microcapsule, and CDP microcapsule.

To examine the effect of molecular geometry of amphiphile on the inner self-assembled structure of the polymer microcapsule, two types of PMMA microcapsules were prepared, as shown in Figure 3a. The proposed amorphous ceramide microcapsule containing both cylindrical ceramide and conical DLGL amphiphiles (CDP microcapsule) is compared with the PMMA microcapsule including only ceramide molecule (CP microcapsule). The microscale morphologies of the prepared microcapsules and pristine ceramide were investigated by field-emission scanning electron microscopy. As illustrated in Figure 3b, the large-sized crystalline aggregates with an average size of 50 µm were observed for pure ceramide due to the strong regular packing of cylindrical amphiphiles in the liquid medium. In contrast, the relatively small-sized spherical morphology was found in CDP and CP microcapsules. Notably, CDP microcapsule with both ceramide and DLGL (25 µm) appears to have a larger particle size than CP microcapsule (15 µm) owing to the additional incorporation of DLGL molecules into the core region of the microcapsule.

A structural analysis of the CDP and CP microcapsules was carried out by Fourier-transform infrared spectroscopy (Figure 3c). The pristine ceramide and DLGL amphiphiles showed several characteristic vibrations of amide group due to a similar molecular structure. The amide I and II bands corresponding to C=O stretching vibration and N-H bending vibration were found at 1625 and 1550 cm^−1^, respectively. In addition, the broad peak at 3290 cm^−1^ was assigned to the N-H stretching vibration of amide group. The CDP and CP microcapsules synthesized through the in situ polymerization of MMA monomer exhibited characteristic bands of amide group stemming from inner core materials similar to those of the pristine ceramide and DLGL. Moreover, a C=O stretching vibration of acrylate group was observed at 1725 cm^−1^, and the absorbance of C=C double bond at 1630 cm^−1^ was greatly reduced by MMA polymerization. These results confirm that hierarchical ceramide microcapsules containing the interpenetrated ceramide and DLGL as core materials were successfully prepared using facile doping of DLGL amphiphile and subsequent in situ polymerization of PMMA in the core-shell geometry. In comparison between CDP and CP microcapsules, the increased absorbance at 1550 cm^−1^ was observed for CDP microcapsule due to the stronger N-H bending vibration of DLGL dopant, indicating the efficient encapsulation of both ceramide and DLGL amphiphiles.

To investigate the existence and structural stability of inner core materials in the hierarchical PMMA microcapsules, thermogravimetric analysis was conducted on CDP and CP microcapsules with different core materials. While the pristine ceramide maintained the initial weight even at 360 °C due to the highly ordered crystalline structure, an abrupt weight loss at 105 °C was observed for pure DLGL molecules with a spherical self-assembled structure (Figure 3d). In the case of CP microcapsule including only ceramide, a noticeable weight loss was exhibited at the reduced temperature of 290 °C compared to that of pure ceramide, suggesting that the confined microcapsule geometry can suppress a high degree of crystallization of inner ceramide molecules. Furthermore, the proposed amorphous CDP microcapsule containing ceramide and DLGL exhibited a large weight loss at the relatively low temperature of 175 °C, indicating the formation of a structurally unstable self-assembled phase in the core region. These results reveal that the cylindrical ceramide and conical DLGL amphiphiles as core materials can cooperatively self-assemble to yield an irregular and unstable architecture of ceramides in the confined microcapsule.

**Figure 4:**
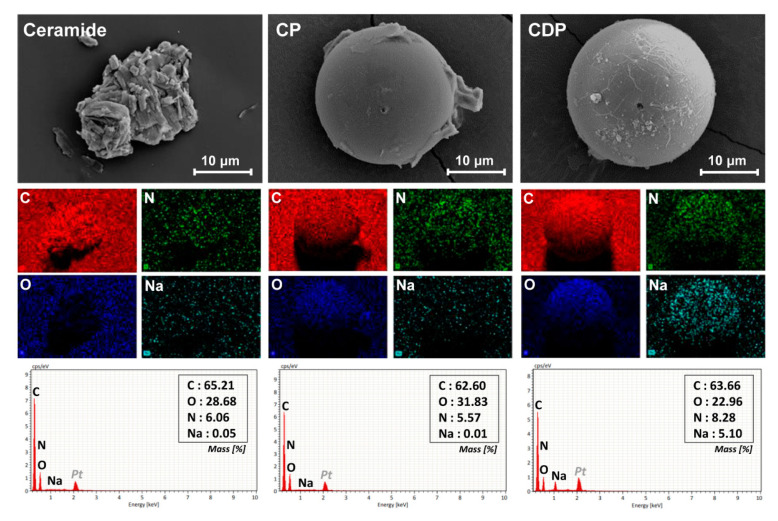
FE-SEM images, EDS mapping images, and EDS spectra of pristine ceramide, CP microcapsule, and CDP microcapsule.

Energy dispersive X-ray spectroscopy was carried out to confirm the elemental composition and distribution of the hierarchical amorphous ceramide microcapsule, as shown in Figure 4. Since the pristine ceramide material consists of carbon, nitrogen, and oxygen atoms, its crystalline aggregate exhibited the reasonable elemental composition and shape-dependent distribution of the corresponding element. In the CP microcapsule including only ceramide, a relatively high content of oxygen element was observed owing to the PMMA shell layer, and Na element corresponding to DLGL molecule was not found in EDS spectrum. On the contrary, hierarchical amorphous CDP microcapsule containing both ceramide and DLGL molecules showed a distinct spherical distribution of Na element in the whole capsule area, indicating the uniform distribution of DLGL amphiphile in the inner self-assembled structure. Therefore, EDS mapping analyses confirm that the DLGL amphiphiles are successfully incorporated into the core of hierarchical ceramide microcapsule and can construct the cooperatively self-assembled amorphous phase along with ceramide molecules.

### 3.2. Morphological Properties of Hierarchical Amorphous Ceramide Microcapsule

**Figure 5:**
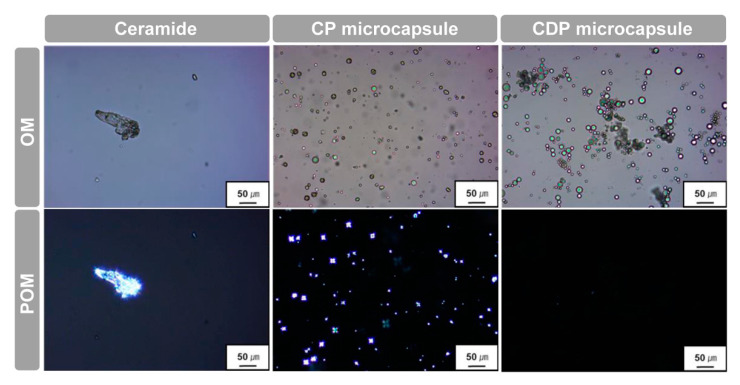
OM and POM images of pure ceramide, CP microcapsule, and CDP microcapsule in deionized water.

In order to examine the morphological characteristics of hierarchical ceramide microcapsules dispersed in the liquid medium, optical microscopic measurements were conducted under parallel or crossed polarizers. Figure 5 shows the OM and POM images of pristine ceramide, CP microcapsule, and CDP microcapsules in deionized water. As mentioned in FE-SEM analysis, pure ceramide exhibited large phase-separated aggregations with a particle size of more than 100 µm and spherulitic morphology under crossed polarization, indicating the formation of highly ordered lamellar crystals in an aqueous medium [26]. On the contrary, the encapsulated ceramides with small mononuclear microcapsule morphology were located in the inner domain of each CP microcapsule, suggesting that the microcapsules were composed of a ceramide core and a PMMA shell. While the CP microcapsule containing ceramide as an inner material showed small spherulites stemming from the restricted lamellar packing of cylindrical amphiphiles only in the core region of microcapsule, the proposed hierarchical amorphous CDP microcapsule including both ceramide and DLGL exhibited a fully dark state in the entire area under the crossed polarizers, suggesting the construction of a completely amorphous phase in the confined microcapsule geometry. Notably, stable PMMA microspheres with a large amorphous nucleus were observed for hierarchical CDP microcapsules due to the effective encapsulation and simultaneous cooperative packing of two amphiphiles inside the PMMA shell. These results suggest that the sodium dilauramidoglutamide lysine as an amphiphilic dopant can form an irregular supramolecular structure via adaptive hydrophobic–hydrophilic interactions in the confined PMMA microcapsule, thereby offering the completely amorphous phase of ceramides.

**Figure 6:**
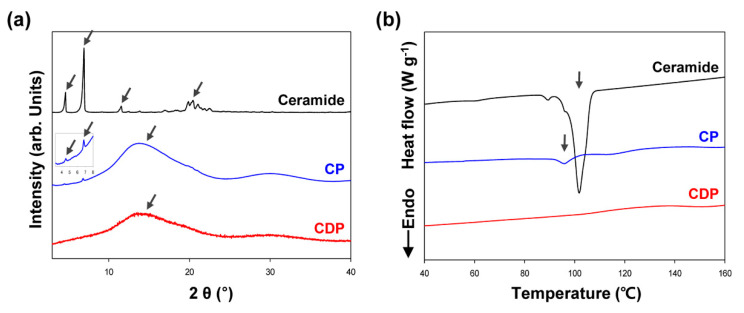
(**a**) XRD patterns and (**b**) DSC thermograms of pure ceramide, CP microcapsule, and CDP microcapsule.

To investigate the structural difference in the self-organized phases of amphiphilic molecules, X-ray diffraction (XRD) experiments were performed on the pristine ceramide, CP microcapsule, and CDP microcapsule (Figure 6a). In the XRD pattern of pure ceramide, a large number of sharp characteristic peaks were exhibited at 2*θ* = 4.59°, 6.93°, 11.54°, 19.87°, 20.47°, and 21.07°, which are attributed to the lamellar crystalline structure of self-assembled cylindrical ceramide molecules [26]. On the contrary, the X-ray pattern of CP microcapsule including only ceramide as a core material showed the low-intensity characteristic peaks corresponding to crystalline ceramide molecules at 2*θ* = 4.59° and 6.93° in the small-angle region and only a broad halo centered at 2*θ* = 15° corresponding to the disordered hydrophobic alkyl chains in the wide-angle region, indicating that the ceramide amphiphiles surrounded by PMMA shell cannot construct the highly ordered crystalline phase with a long-range order due to the confinement effect of the microcapsule. The encapsulation of crystalline ceramides inside the PMMA microcapsule hindered their crystallization and therefore afforded a less-ordered supramolecular structure. The similar XRD results have been reported on the capsule systems including crystalline curcumin materials [28,29]. In the XRD pattern of CDP microcapsule, the proposed hierarchical PMMA microcapsule containing both ceramide and DLGL exhibited no characteristic peaks of the pure crystalline ceramide in the small-angle region and only a broad band similar to that of CP microcapsule in the wide-angle region, which reflects the formation of an irregular amorphous structure in the inner core region. After the employment of conical DLGL dopant into the internal domain of cylindrical ceramides during PMMA microencapsulation, the crystalline property of pristine ceramide disappeared, and the self-constructed amorphous phase with an irregular packing structure was newly formed inside the microcapsule.

Thermal phase transitions and crystallinity of pure ceramide and hierarchical ceramide microcapsules were studied by the differential scanning calorimetry. Figure 6b shows the DSC thermograms of pristine ceramide, CP microcapsule, and CDP microcapsule on heating. The phase transition behavior of hierarchical ceramide microcapsules was different from that of the pure ceramide. While pristine ceramide showed a strong endothermic peak at 102.6 °C corresponding to the melting transition [30], CP microcapsule exhibited a weak melting peak at 96.3 °C. Notably, CP microcapsule showed a relatively small enthalpy value of melting transition (2.5 J g^−1^) compared to that of pure ceramide (94.0 J g^−1^), suggesting a lower crystallinity in the internal area of the microcapsule. It is supposed that the low crystallinity of CP microcapsule is ascribed to the confinement of ceramide molecules inside the PMMA microcapsule, and this result is in accord with the POM and XRD observations. In CDP microcapsule, no endothermic peak for pure ceramide was observed in the temperature range of the crystalline phase, which is indicative of a completely disordered amorphous phase. As a consequence, it is confirmed that the proposed approach based on the polymeric microencapsulation of cylindrical ceramide with conical DLGL dopant can afford a completely amorphous structure of conventional ceramide material.

### 3.3. Long-Term Stability of Hierarchical Amorphous Ceramide Microcapsule

**Figure 7:**
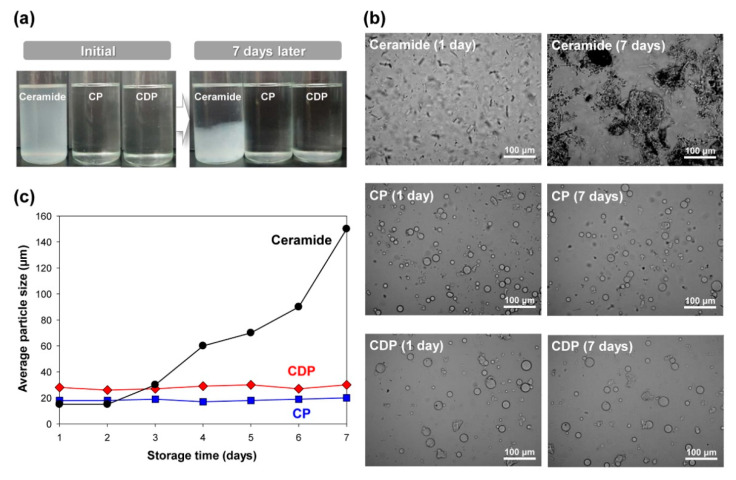
(**a**) The macroscopic and (**b**) microscopic optical images of pristine ceramide, CP microcapsule, and CDP microcapsule in mineral oil after initial dispersion and seven days. (**c**) Dependence of average particle size on storage time for pure ceramide, CP microcapsule, and CDP microcapsule.

To examine the effect of polymeric microencapsulation on the long-term physical stability of ceramides, which is an essential attribute of controlled release system for epidermal barrier application, dispersibility test was performed in mineral oil at 25 °C for seven days after mechanical agitation for 1 h. Figure 7a,b show the macroscopic and microscopic optical images of pristine ceramide, CP microcapsule, and CDP microcapsule after initial dispersion and seven days. For pure ceramide, a cloudy and unstable suspension state occurred immediately after initial agitation, and severe phase separation of ceramides was observed after seven days, as shown in Figure 7a. Contrarily, CP and CDP polymer microcapsules exhibited the clear appearance of the ceramide microcapsule dispersions and maintained it even after seven days. While the significant agglomeration of ceramide particles was observed for pristine ceramide, the PMMA microcapsule systems containing ceramide or DLGL as a core molecule maintained the particle shape and size distribution even after seven days, as shown in Figure 7b. Since the Brownian motion plays a key role in the dynamics of tiny particles, active drift motion of ceramide particles induces particle agglomeration through flocculation, resulting in the long-term instability of ceramide suspension [31]. On the contrary, hydrophobic PMMA shell can stabilize the dispersion state of polymeric microcapsules including ceramide molecules in the mineral oil via hydrophobic interactions, leading to the improvement in the physical stability of the proposed hierarchical amorphous ceramide microcapsules.

The particle size analysis supported the long-term stability of hierarchical amorphous ceramide microcapsules, as shown in Figure 7c. Even after seven days, the CP and CDP polymer microcapsules exhibited the constant particle sizes of fine microcapsules with average diameters of 19 and 28 μm, respectively. On the contrary, as the storage time of pure ceramide increased, the average particle size was increased, strongly indicating the existence of considerable particle agglomeration during seven days. Therefore, the proposed fabrication strategy for completely amorphous hierarchical ceramide microcapsule using polymer encapsulation and DLGL dopant can afford an excellent dispersibility in the liquid medium as well as the long-term physical stability of amorphous ceramide for a potential epidermal barrier.

## 4. Conclusions

In summary, a facile yet effective, scalable strategy to fabricate completely amorphous and highly sustainable hierarchical ceramide microcapsules for a potential epidermal barrier has been newly developed using the polymeric encapsulation of the interpenetrating conical amphiphiles with a different critical packing parameter. Even though the amphipathic cylindrical ceramide as a bioactive lipid plays a crucial role in constructing an epidermal barrier with a robust bilayer architecture, there is no precedent for the fabrication of a completely amorphous ceramide structure for efficient penetration into the epidermis. The elegant hybrid concept using polymeric microencapsulation and critical packing parameter is applied to the first successful attempt to prepare the self-assembled amorphous architecture of ceramides in the confined polymer microcapsule. To attain a completely amorphous morphology of ceramides, sodium dilauramidoglutamide lysine as a conical amphiphilic dopant with a different critical packing parameter was incorporated into the core of PMMA microcapsule containing ceramides. The specific hydrophobic–hydrophilic interactions between cylindrical ceramides and conical amphiphiles induced an irregular packing structure in the restricted core region of the polymeric microcapsule, and consequently, endowed the hierarchical ceramide microcapsule with a completely amorphous structure after polymeric microencapsulation. This unprecedented approach rendered it possible to achieve the long-term physical stability of amorphous ceramide microcapsule with an excellent dispersibility in the liquid medium compared to common crystalline ceramides. Furthermore, the self-constructed amorphous ceramide microcapsule exhibited excellent thermal durability up to 175 °C in high-temperature conditions. Notably, the proposed hierarchical amorphous ceramide microcapsule could be simply prepared in large quantities at low cost through simple suspension polymerization without the need of complex synthetic procedures. It is believed that the proposed approach opens a new way to prepare functional amorphous bioactive materials with potential applications, such as epidermal barrier, controlled-release microsystem, drug delivery vehicle, and biosensor.
